# Effect of an osteoporotic fracture prevention program on fracture incidence in routine care: a cluster-randomized trial

**DOI:** 10.1186/s12916-021-02226-8

**Published:** 2022-02-04

**Authors:** Kilian Rapp, Sarah E. Lamb, Patrick Roigk, Clemens Becker, Claudia Konnopka, Hans-Helmut König, Raphael S. Peter, Dietrich Rothenbacher, Gisela Büchele

**Affiliations:** 1 Department of Clinical Gerontology, Robert-Bosch-Hospital, Auerbachstr. 110, 70376 Stuttgart, Germany; 2 College of Medicine and Health, St Lukes Campus, University of Exeter, Exeter, EX12LU UK; 3 Department of Health Economics and Health Services Research, University Medical Center Hamburg-Eppendorf, Hamburg, Germany; 4 Institute of Epidemiology and Medical Biometry, Ulm University, Helmholtzstr. 22, 89081 Ulm, Germany

**Keywords:** Falls, Osteoporotic fractures, Prevention, Rural area, Mobility and falls prevention exercise classes, DXA

## Abstract

**Background:**

Fractures are a major health problem in aging societies. Preventive approaches combining bone health and fall prevention are rare. The osteoporotic fracture prevention program in rural areas (OFRA) is a health care fund-driven program for older people in randomly selected districts in Germany. The components of the program were falls prevention exercise classes, examination of bone health by a dual-energy X-ray absorptiometry (DXA) scan, and a consultation about “safety in the living environment.” The aim of this study was to evaluate this complex preventive intervention in a routine health care setting.

**Methods:**

This cluster-randomized trial was performed from October 2015 to October 2018 and took place in 186 administrative districts in five federal states, 47 districts served as intervention districts, and 139, as controls. Within these districts, we included (a) all community-living women and men aged 70–85 years with prior fragility fractures and (b) all community-living women aged 75–80 years. The analysis used routine data collected by a health insurance company. The primary endpoint was all fragility fractures combined. Fracture types, mortality, and nursing home admission were explorative endpoints. Cox frailty models were used for comparative analyses with a median follow-up time of 365 days (interquartile range: 0 days).

**Results:**

Nine thousand four hundred eight individuals were approached to participate in one of the program components, 27,318 individuals served as controls. The mean age was 78.7 years. Of those approached to participate, nearly 30% joined the exercise classes. DXA measurement was reimbursed for 13.6%, and 51.8% received advice about measures to increase “safety in the living environment.” The incidence of fragility fractures did not differ between the intervention and the control group (HR 0.94; 95% CI 0.80–1.11). However, femoral fractures, the most frequent fracture type, were reduced in the intervention group (HR 0.76; 95% CI 0.59–0.99). Mortality and nursing home admission did not differ between the intervention and the control group.

**Conclusions:**

A comprehensive fracture prevention program for older people living in rural areas was implemented. The program did not affect the primary endpoint of all fragility fractures combined. It has to be considered that we used a modified intention to treat approach based on geographic randomization and information about endpoints relied exclusively on routine data of the health care insurance.

**Trial registration:**

German Clinical Trials Register DRKS-ID: 00009000

**Supplementary Information:**

The online version contains supplementary material available at 10.1186/s12916-021-02226-8.

## Background

Fractures are a major health problem in aging societies undergoing demographic transition [1]. Functional impairment, psychological problems including anxiety or depression, and the disruption of the personal social infrastructure are frequent sequelae of fractures and among the primary reasons for disability and loss of autonomy in older people. Femoral fractures are the most common, costly, and resource-consuming type of fragility fractures in Germany [2].

The two main underlying mechanisms of fragility fracture are osteoporosis and falls [3, 4]. For community-living people, there is a large body of evidence that physical exercise with components of strength and balance training is the most effective measure [5]. The modification of environmental hazards is an additional approach to reduce fall risk [6].

Several pharmaceutical agents offer effective treatment options for the reduction of fragility fractures [3, 7]. Pharmaceutical management is usually restricted to people with osteoporosis which account only for a limited proportion of older people with increased fracture risk. Therefore, coordinated preventive approaches are needed which combine bone health and fall prevention in order to reduce the burden of falls and fall-related fractures [8].

In most western countries, fall prevention offerings for community-living people are limited and not well coordinated. The availability of exercise classes in rural areas is particularly problematic. People from these areas are usually confronted with long and unacceptable distances to travel to attend an exercise class. Furthermore, bone health is a neglected field in many countries in the world [9, 10].

Coordinated preventive approaches which combine bone health and fall prevention as part of routine care in primary and secondary fracture prevention are rare and randomized trials are also not available so far.

The osteoporotic fracture prevention program in rural areas (OFRA) was a large health care fund-driven program for older people conducted as part of routine care in randomly selected districts in Germany. The program was delivered as a cluster-randomized trial in rural areas and aimed to improve physical function and reduce the risk of falls and fractures. The components of the program were mobility and falls prevention exercise classes, the examination of bone health by a DXA scan, and a consultation about “safety in the living environment.” The aim of this study was to evaluate this complex preventive intervention in a routine health care setting.

## Methods

OFRA (German: ‘Trittsicher durchs Leben‘) is a large health care fund-driven program to improve safe mobility and reduce the risk of falls and fractures in older people living in rural areas. The health care fund (Sozialversicherung für Landwirtschaft, Forsten und Gartenbau - SVLFG) covers 1.6% of the German population aged 70 years and older and is compulsory for people working in agriculture, gardening, and forestry. Therefore, the study included only individuals who have been or are still working in agriculture, gardening, and forestry. For OFRA the health care fund cooperates with the “German Association of Rural Women” (LandFrauenverband - dlv), a volunteer organization of women living in rural areas, the German Gymnastics Association (Deutscher Turnerbund - DTB), and the Robert Bosch Gesellschaft für Medizinische Forschung, Stuttgart (RBMF). Details can be found in the study protocol [11].

### Intervention areas/randomization

**Fig. 1:**
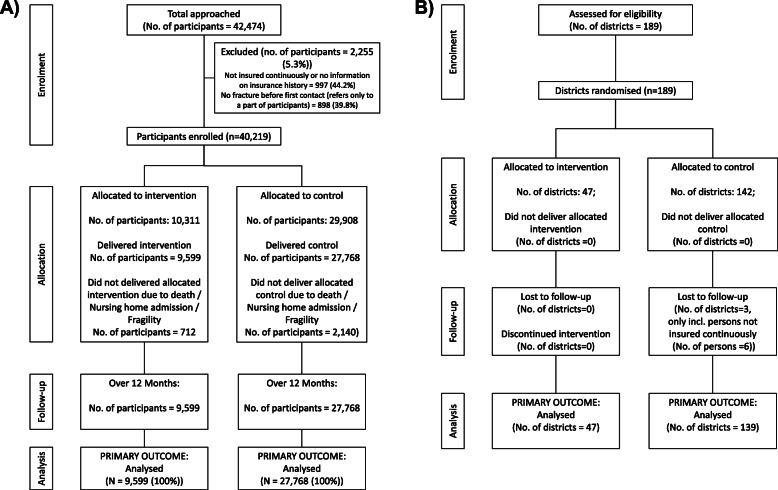
Flow-chart of **A** the number of included districts (cluster of randomization) and **B** the number of included individuals

The implementation of the program took place in 47 administrative districts in five federal states (i.e., Baden-Württemberg, Bavaria, Hesse, Lower Saxony, Rhineland-Palatinate). These federal states cover a large percentage of the whole area of Germany. All administrative districts of the five federal states were randomly assigned to either the implementation or control group (cluster randomization; 47 intervention districts, 139 control districts). Randomization was stratified by federal state. The control districts did not receive any intervention within the program (Fig. 1A).

### Included individuals

The program was offered as a new component of routine care to (a) all community-living women and men aged 70 to 85 years with a history of a fragility fracture (femur, spine, forearm, shoulder/upper arm, lower leg, or pelvis) in the previous 5 years and (b) all community-living women aged 75 to 80 years who lived in the intervention districts and were insured by the SVLFG (complete sampling). The choice of these two subgroups and their age ranges followed different considerations: (a) to meet the recommendations of the German osteoporosis guideline for bone health evaluation (https://dv-osteologie.org/osteoporose-leitlinien), (b) to capture people with a high imminent fracture risk, and (c) to combine the complete sampling approach with the staff capacities of the health care fund. Insured individuals were excluded if they lived in a care home or if their formal home care need for the basic activities of daily living was 120 min or more according to the categorization of the German long-term care insurance (level of care: grade 2 or 3). Individuals of the intervention and control districts were identified from the health insurance records in August 2015 and then subsequently recruited and entered into the study and contacted about the interventions over a 2-year period (1 October 2015–30 September 2017). If one of the exclusion criteria or death occurred before study entry, the selected individual was excluded. The staggered study entry was needed to manage the resource available to provide the intervention. Insured individuals aged 70 to 85 years who were not selected in August 2015 but had an incident fragility fracture during the recruitment period were additionally included. Inclusion and exclusion procedures were handled identically for individuals from the intervention and control districts.

### Intervention program

The program had 3 components: 1)  ﻿“Trittsicher”-mobility and falls prevention exercise classes, 2)  the examination of bone health by a bone mineral density measurement (dual-energy X-ray absorptiometry (DXA) scan), and 3) a﻿ consultation about “safety in the living environment.” Details are described in Table 1 and in the study protocol [11].

**Table 1:** Details of the intervention program

Components	Details
“Trittsicher”-mobility and falls prevention exercise classes	• Were based on the Otago exercise program [12, 13] and a program developed for groups by the German Gymnastics Association [14] • Had 6 sessions à 90 min delivered within 6 weeks • Wanted participants to perform exercises at home between and after the end of the group sessions (booklet with instructions and a training log) • Took place in local facilities to keep the distances as short as possible • Were usually organized by the “German Association of Rural Women” (LandFrauen) • Were free of charge
Bone mineral density measurement by dual-energy X-ray absorptiometry (DXA)	• Was recommended to all individuals • Was based on the German osteoporosis guideline which recommends a baseline assessment for bone health including a DXA scan in all women aged 70 and more (http://dv-osteologie.org/osteoporose-leitlinien) • Was reimbursed by the program independently from a fracture history • Individuals were asked to talk with their general practitioners (GP) about the need for a DXA scan. GPs received a one-time payment for counseling and handling potential consequences like treatment of a diagnosed osteoporosis
Safety in the living environment	• Individuals were advised by a “prevention manager” of the agricultural accident insurance (part of the SVLFG) how to increase safety in the living environment if required (e.g., by installation of handrails at the entrance or better lighting on paths around the farm still used by the participants)

The selected individuals in the intervention districts were informed about the program by a letter by the health care fund and visited one to four weeks later at home by a “prevention manager” who motivated them to attend an exercise class or to make use of a DXA measurement. If required, recommendations to increase safety in the nearby living environment were given. The prevention manager contacted the insurance telecentre and informed them of the individual’s willingness to attend exercise sessions. The telecentre referred individuals who were willing to attend exercise sessions to a class taking place near their home and informed them about DXA devices in the surrounding area. Individuals received a final report in which their preferences regarding the components of the program, dates, and arrangements were summarized and information sheets for the GP were included.

One of the objectives of OFRA was to build up an infrastructure of “Trittsicher”-mobility and falls prevention exercise classes. About 700 exercise instructors received a mandatory training course in the year before the start of recruitment. The exercise classes were generally free of charge and also open to older people who were not selected for the scientific program. During the 2-year recruitment and 1-year follow-up period, 2339 “Trittsicher”-mobility and falls prevention exercise classes with a mean number of 11.6 participants per class were implemented in the intervention districts [15].

### Outcome measures

The primary outcome was a measure of incident fragility fractures (ICD-10 S12, S22, S32, S42, S52, S72, S82) requiring hospitalization and recorded as main diagnosis in the routine health claims database of the insurance company after study entry. Not à priori defined explorative endpoints were the fractures of each entity separately (femur (S72), spine (S12, S22.0, S22.1, S22.2, S22.3, S22.4, S22.5, S22.8, S22.9, S32.0, S32.7, S32.82), forearm (S52), shoulder/upper arm (S42), lower leg (S82), pelvis (S32.1, S32.2, S32.3, S32.4, S32.5, S32.81, S32.83, S32.89)), death, and nursing home admission. Uptake of a “Trittsicher”-mobility and falls prevention exercise class, reimbursed DXA measurements, new prescriptions of specific anti-osteoporotic drugs, and advice given about measures to increase “safety in the living environment” served as process parameters.

### Data source

The study database was merged with the routine data collection system of the health insurance company to gain data on incident fractures, reimbursed DXA measurements, new prescriptions of specific anti-osteoporotic drugs (bisphosphonates and denosumab; ATC-codes: M05BA, M05BB, M05BX) prescribed after discharge and dispensed by community pharmacists, nursing home admission, and death. Information about uptake of a “Trittsicher”-mobility and falls prevention exercise class was derived from a telephone interview of a random subsample of 780 selected individuals of the intervention districts since this information was not available in the routine data collection system [16]. The information on advice given to individuals on “safety in the living environment” was extracted from the records of feedback from the prevention managers to the telecentres.

### Study entry, matching procedure, and follow-up

The date of study entry of the individuals of the intervention group was defined by the date of a first information letter sent by the health insurance company. Since the letter was usually followed by a visit of a prevention manager, a recruitment period of 2 years was needed. To align data collection periods between the intervention and control groups and to receive a “study entry date” for the control-persons, for each individual in the intervention arm we identified people in the control districts to provide data for the equivalent time period. Federal state and target group (women and men 70 to 85 years with prior fracture; women 75 to 80 years) were used as matching criteria, resulting in 10 strata. Within each stratum, for each individual of the intervention districts, an average of three individuals from the control districts (according to the randomization balance) was matched randomly with replacement. This procedure was performed for individuals selected in August 2015 and for those with an incident fragility fracture during the recruitment period in the same way. The SAS-PROC SURVEYSELECT was used to perform the matching procedure.

The time-period between the information letter and the start of an exercise class was usually 2 months or more. Therefore, follow-up began 2 months after the date of the letter in the individuals of the intervention districts (independently if they participated in an exercise class or not) and 2 months after the assigned study entry in the matched individuals of the control districts and was 12 months or until censoring due to a fracture death or study ending. In an additional sensitivity analysis, the maximal available follow-up time of each included individual was used which is one up to 3 years (in median 1.7 years).

### Statistical methods

All selected individuals from the intervention districts and the matched individuals from the control districts were included in the analyses. This means that individuals from the intervention districts who did not receive any component of the intervention were also part of the analyses (modified intention to treat approach). Cox frailty analyses were used to calculate hazard ratios (HR) and 95% confidence intervals (95% CI). All models included federal state in the strata statement to account for the stratified randomization. To account for the cluster-randomized design of the study, a random effect-statement including the administrative districts was applied. No further adjustment was performed. Since the intervention did not influence mortality, death was not considered as competing risk. The proportional hazard assumption was checked applying proportionality tests and by inspection of Schoenfeld-residuals and found to be fulfilled for all models. Rates per 1000 person-years were calculated for the total study population and stratified by sex, age, and a history of fracture. All calculations were performed using SAS 9.4.

The study protocol was published in 2016 [11]. The study was registered at the German Clinical Trials Register (DRKS-ID: 00009000) and approved by the ethics committee of Ulm University (proposal 120/15).

## Results

In the intervention group, 9408 insured individuals from 47 districts were approached; 27,318 insured individuals from 139 districts served as controls (Fig. 1). Characteristics of individuals in intervention and control districts were similar. Nearly 90% were women, the mean age at study entry was 78.7 years, about 30% had a fracture history in the 5 years before study entry, and more than 8% had a specific anti-osteoporotic drug prescription in the 6 months before study entry (Table 2).

**Table 2:** Baseline characteristics of the matched individuals in the intervention and control districts

	Intervention districts	Control districts
Number of districts, *N*	47	139
Number of individuals per district, median (Q1–Q3)	186 (106–276)	168 (107–256)
Number of individuals in the study, *N*	9408	27,318
Federal states		
Baden-Württemberg, *n* (%)	1513 (16.1%)	4534 (16.6%)
Bavaria, *n* (%)	4351 (46.2%)	11,974 (43.8%)
Hesse, *n* (%)	593 (6.3%)	2171 (7.9%)
Lower Saxony, *n* (%)	1971 (21.0%)	6224 (22.8%)
Rhineland Palatinate, *n* (%)	980 (10.4%)	2415 (8.8%)
Characteristics of the individuals		
Women, *n* (%)	8414 (89.4%)	24,566 (89.9%)
Age at study entry, mean (SD)	78.8 (2.5)	78.8 (2.5)
Fracture history before study entry*, *n* (%)	2834 (30.1%)	8017 (29.3%)
No care need at study entry^†^, *n* (%)	8371 (89.0%)	24,054 (88.1%)
Specific anti-osteoporotic drug prescriptions before study entry^‡^, *n* (%)	835 (8.9%)	2248 (8.2%)
Follow-up time: Number of individuals with one year of follow-up, *N* (%)	8070 (85.8%)	23,474 (85.9%)
Time in days, median (Q1–Q3)	365 (365–365)	365 (365–365)

*SD* standard deviation, *N*, number of individuals, *Q1–Q3* 1st and 3rd quartile

*Femur, spine, forearm, shoulder/upper arm, lower leg, and pelvis fracture in the time period of 5 years before study entry

^†^Care need is defined according to the categorization of the German long-term care insurance

^‡^Specific anti-osteoporotic drug prescription dispensed by community pharmacists in the time period of 6 months before study entry

Nearly 30% of the approached individuals participated in an exercise class, and 51.8% received advice about measures to increase “safety in the living environment.” DXA- measurements were reimbursed, and new specific anti-osteoporotic drugs were prescribed in 13.6% and 3.8% of the approached individuals in the intervention group and in 1.6% and 1.9% in the individuals in the control group, respectively (Tabl﻿e 3).

**Table 3:** Program components and process parameters in the intervention and control group

Program components and process parameters	Data source	Intervention group ***N*** (%)	Control group ***N*** (%)
Uptake of a “Trittsicher”- mobility and falls prevention exercise class	Telephone interview of a random subsample of *n* = 780 individuals of the intervention group (Roigk et al. 2020 [16])	231 (29.6)	n.a.
Reimbursed DXA- measurements	Routine data of the health insurance company (*n* = 36,726); see the “Methods” section	1277 (13.6)	426 (1.6)
New prescriptions of specific anti-osteoporotic drugs*	Routine data of the health insurance company (*n* = 36,726); see the “Methods” section	358 (3.8)	517 (1.9)
Advice given about measures to increase “safety in the living environment”	Feedback from the prevention managers to the telecentres after visit of the individuals of the intervention group	4869 (51.8)	n.a.

n.a., not applicable; *N*, *n* = number; *bisphosphonates, denosumab

**Fig. 2:**
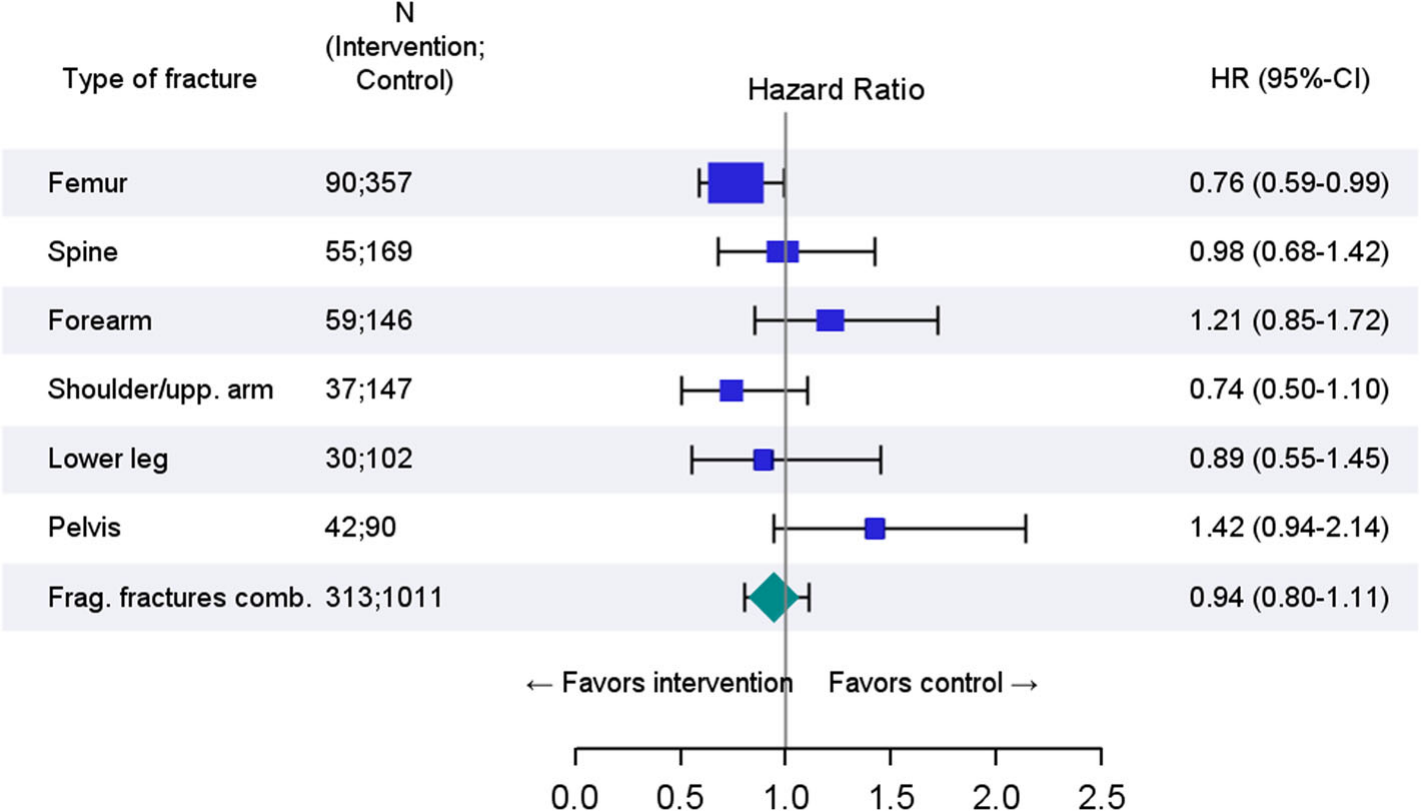
Effect of OFRA on all “fragility fractures combined” and on fractures of femur, spine, forearm, shoulder/upper arm, lower leg, and pelvis during 12 months of follow-up. *N*, number; HR, hazard ratio; CI, confidence interval; Frag. Fractures comb., fragility fractures combined

The incidence of the primary endpoint “fragility fractures combined” did not differ between the intervention and the control group (hazard rate (HR) 0.96; 95% CI 0.82–1.13) (Fig. 2). The most frequent fracture type was femoral fractures (Additional file 1: Table A). In comparison to the control group, the rate of femoral fracture was significantly reduced in the intervention group (HR 0.76; 95% CI 0.59–0.99). A statistically non-significant tendency was observed in shoulder/upper arm fractures in favor of the intervention and in pelvic fractures in favor of the control. In the sensitivity analyses with the maximal available follow-up time for each included individual (1 year up to 3 years), the risk for the primary endpoint “fragility fractures combined” changed slightly to a HR of 1.04 and was statistically still not significant (95% CI 0.91–1.19).

**Fig. 3:**
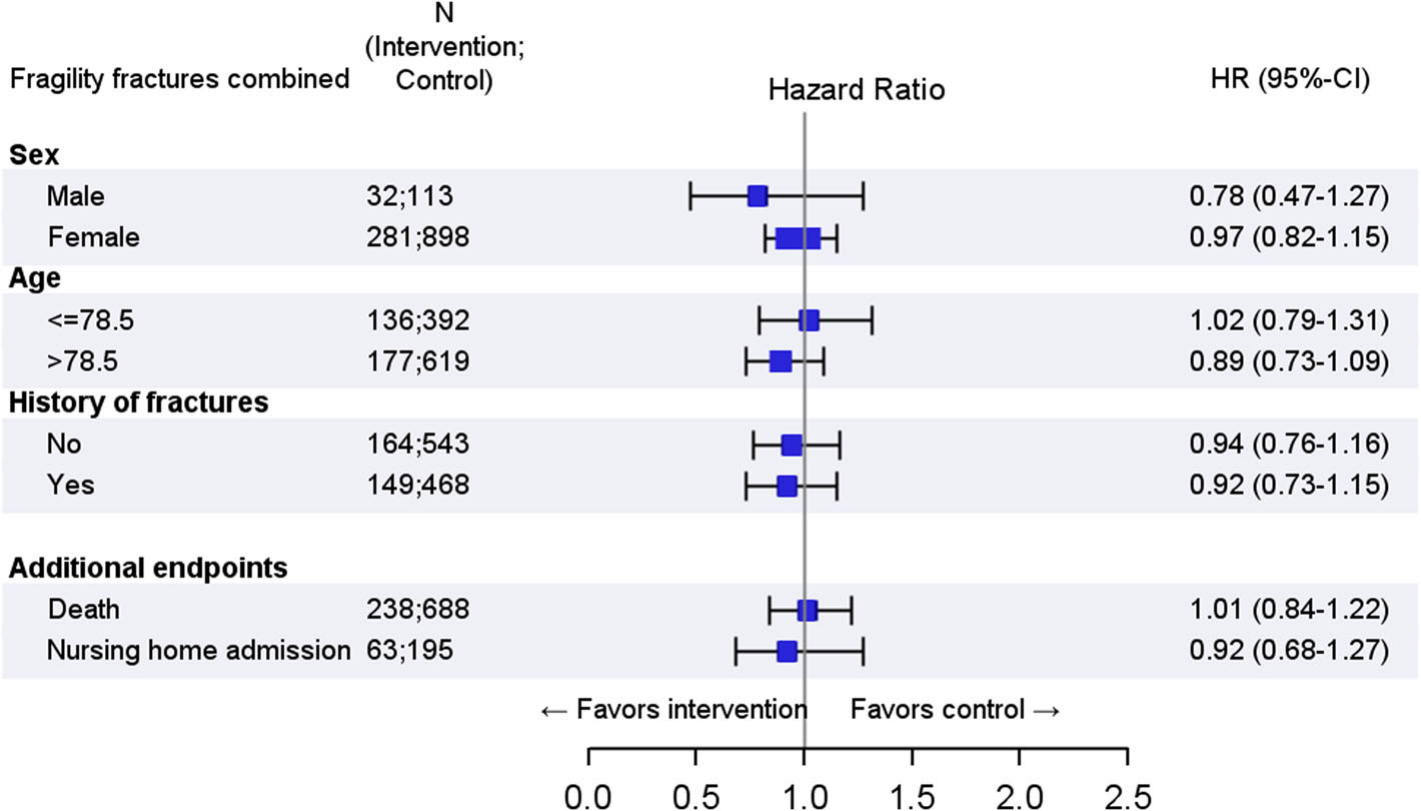
Effect of OFRA on all “fragility fractures combined” stratified by sex, age, and fracture history and on death and nursing home admission during 12 months of follow-up. *N*, number; HR, hazard ratio; CI, confidence interval; FU, follow-up

The stratified analyses by sex, age, and fracture history did not show a statistically significant advantage of the intervention in one of the strata on fragility fractures combined (Fig. 3; Additional file 1: Table B). Mortality and nursing home admission did not differ between the intervention and the control group.

## Discussion

We analyzed a complex health insurance fund-driven intervention which was performed as a cluster-randomized trial in a routine health care setting. The intervention neither reduced the primary endpoint “fragility fractures combined” in our analysis with an identical follow-up time in all individuals nor in our sensitivity analysis with a maximal available but different follow-up time for each individual. However, femoral fractures, the most important fracture type in old people regarding frequency, consequences, and health care costs, were reduced by 24%. Mortality or nursing home admission was not reduced by the intervention.

We do not know of a similar approach which combined different components of fracture prevention in a routine health care setting by a randomized trial. There is only limited evidence from meta-analyses [5, 17] but not from single trials [18] and even less from health insurance or healthcare-based programs that exercise is able to reduce fractures.

Systematic approaches to tackle osteoporosis like a fracture liaison service (FLS) or disease management programs have been initiated and shown that they are able to improve drug treatment in primary and secondary prevention [19–21]. However, there exist no randomized trials which have demonstrated a beneficial effect of these approaches on fracture incidence.

Coordinated preventive approaches which combine bone health and fall prevention either in trials or in routine care for primary and secondary prevention are extremely rare. A program from a health maintenance organization in Southern California approached their members at risk actively and addressed not only bone health but also fall risk [22]. The evaluation of this program was not based on a randomized trial but on a historical control group and suggested a reduction of femoral fractures.

A problem of outcome evaluation of such programs is that they need to be sufficiently powered to demonstrate a significant effect on fracture risk. An answer may come from large simple trials like OFRA embedded in routine health care. In such studies, the implementation is usually not as complete and the compliance not as accurate as in clinical trials, but better reflecting real-life aspects and routine practice. In OFRA, the cooperation of stakeholders from rural areas allowed implementation a large number of exercise classes within a short period which was continued after the end of the scientific phase of the program.

Information who attended an exercise class was not available in the routine claims data. However, this information was derived by telephone interviews in a random subsample of 780 individuals [16]. The uptake of an exercise class was nearly 30% in the approached individuals in the intervention group, more than half participated in all six class sessions, and 59% stated of having continued with the home-based exercise after the end of the classes. Individuals could attend up to two exercise classes per year. Many individuals made use of it, but we have no information about the exact percentage. The use of a bone mineral density measurement and the initiation of an antiosteoporotic medication was clearly lower. There is no information which percentage of the given recommendations about safety in the living environment was realized. We think that particularly the uptake rate of an exercise class is respectable; nevertheless, the majority of the selected individuals in the intervention districts did not receive one or more of the offered components. Since we included all selected individuals in our analyses independently if they received one of the intervention components (modified intention to treat approach based on district randomization), an attenuation of the effect of the intervention has to be expected.

We do not know the exact mechanism that our program had on femoral fracture incidence. It is interesting that the finding on femoral fractures is similar to a trial of osteoporosis screening and medication in primary care [23]. This trial found no effect on overall fragility fractures but a beneficial effect on femoral fractures.

Apart from femoral fractures, we observed statistical non-significant trends in favor of the intervention for fractures of the shoulder/upper arm and to a lesser extent of the lower leg. However, we observed also trends in the opposite direction for fractures of the forearm and particularly of the pelvis. We cannot rule out that persons that participated in the exercise program might have changed their falling directions and landing strategy leading to backward landing position instead of side impact.

The strengths of the study are the large sample, the cluster-randomized design, and the comprehensive anti-fracture approach. It was demonstrated that such a program can overcome the limited infrastructure of rural areas and the otherwise long distances to preventive offerings. In addition, the program may have increased physical activity, social interaction, and wellbeing.

A limitation of our study is that information about fracture incidence relied exclusively on hospitalizations recorded by routine data of the health care insurance. Fractures which did not need hospitalization were not captured. Furthermore, no information about falls or participation in an exercise class (beyond the information from a telephone interview of a random subgroup [16]) was available on an individual basis. The usual design for cluster-randomized trials is to assemble clusters of participants prior to randomization as this reduces important biases. In our analysis, matching was undertaken purely to ensure that the data capture times for treatment and control were contemporaneous. We observed a significant reduction of femoral fractures. However, femoral fracture was only an explorative endpoint. Therefore, the observed significance may be only by chance.

The program was delivered by stakeholders which may be countryside and country specific and received by people who have been working in agriculture, gardening, and forestry and who are living in a rural surrounding. This specific setting limits the generalizability of the approach. However, fracture burden is high and the implementation gap for preventive measures is large in many high-industrialized countries. Therefore, OFRA may serve as a modifiable model for other countries or healthcare systems.

## Conclusions

A coordinated preventive approach combining bone health and fall prevention was embedded in routine care and delivered as cluster-randomized study in rural areas. The intervention reduced the explorative endpoint femoral fractures but not the primary endpoint fragility fractures combined.

## Supplementary Information

**Additional file 1.** Appendix table A and appendix table B: Table A: Effect of OFRA on all ‘fragility fractures combined’, on different types of fractures, on death and nursing home admission during 12 months of follow-up. Table B effect of OFRA on all ‘fragility fractures combined’ and different types of fractures stratified by sex, age, and fracture history during 12 months of follow-up.

## Data Availability

Data cannot be shared publicly because they were originated from the routine data collection system of a health insurance company.

## Abbreviations

OFRA: The osteoporotic fracture prevention program in rural areas
SVLFG: Sozialversicherung für Landwirtschaft, Forsten und Gartenbau
DTB: Deutscher Turnerbund
RBMF: Robert Bosch Gesellschaft für Medizinische Forschung
