# Supplementary material for: Effect of an osteoporotic fracture prevention program on fracture incidence in routine care: a cluster-randomized trial
Source: BMC Med. 2022 Feb 4;20:49. doi: 10.1186/s12916-021-02226-8 (PMC8815238; doi:10.1186/s12916-021-02226-8)
Supplement: Supplementary file 1 — Additional file 1. Appendix table A and appendix table B: Table A: Effect of OFRA on all ‘fragility fractures combined’, on different types of fractures, on death and nursing home admission during 12 months of follow-up. Table B effect of OFRA on all ‘fragility fractures combined’ and different types of fractures stratified by sex, age, and fracture history during 12 months of follow-up. [file 12916_2021_2226_MOESM1_ESM.docx]

**Additional file**

Table A: Effect of OFRA on all ‘fragility fractures combined’, on different types of fractures, on death and nursing home admission during 12 months of follow-up

|  | **Intervention group** | | | **Control group** | | |
| --- | --- | --- | --- | --- | --- | --- |
|  | **Number of fractures** | **Person- years** | **Rates*** | **Number of fractures** | **Person- years** | **Rates*** |
| Fragility fractures combined* | 313 | 9045.3 | 34.6 | 1011 | 26207.0 | 38.6 |
| Fracture of the femur | 90 | 9045.3 | 9.9 | 357 | 26207.0 | 13.6 |
| Fracture of the spine | 55 | 9045.3 | 6.1 | 169 | 26207.0 | 6.4 |
| Fracture of the shoulder/upper arm | 37 | 9045.3 | 4.1 | 147 | 26207.0 | 5.6 |
| Fracture of the forearm | 59 | 9045.3 | 6.5 | 146 | 26207.0 | 5.6 |
| Fracture of the pelvis | 42 | 9045.3 | 4.6 | 90 | 26207.0 | 3.4 |
| Fracture of the lower leg | 30 | 9045.3 | 3.3 | 102 | 26207.0 | 3.9 |
| Death | 238 | 9188.3 | 25.9 | 688 | 26683.9 | 25.8 |
| Nursing home admission | 63 | 9170.2 | 6.9 | 195 | 26611.3 | 7.3 |

*Numbers of fractures per 1000 person-years

Table B: Effect of OFRA on all ‘fragility fractures combined’ and different types of fractures stratified by sex, age, and fracture history during 12 months of follow-up

|  | **Number of fractures**  **(IG; CG)** | **Person-years**  **(IG; CG)** | **Rates**  **(IG; CG)** | **Hazard ratio**  **(95% CI**) |  | **Number of fractures**  **(IG; CG)** | **Person-years**  **(IG; CG)** | **Rates**  **(IG; CG)** | **Hazard ratio**  **(95% CI**) | |
| --- | --- | --- | --- | --- | --- | --- | --- | --- | --- | --- |
| **Sex** | **Men** | | | |  | **Women** | | | |  |
| Fragility fractures combined* | 32; 113 | 931.5; 2553.6 | 34.4; 44.3 | 0.78 (0.47-1.27) |  | 281; 898 | 8113.8; 23653.4 | 34.6; 38.0 | 0.97 (0.82-1.15) | |
| Fracture of the femur | 10; 46 | 931.5; 2553.6 | 10.7; 18.0 | 0.63 (0.28-1.39) |  | 80; 311 | 8113.8; 23653.4 | 9.9; 13.1 | 0.79 (0.60-1.04) | |
| Fracture of the spine | 12; 37 | 931.5; 2553.6 | 12.9; 14.5 | 0.81 (0.35-1.88) |  | 43; 132 | 8113.8; 23653.4 | 5.3; 5.6 | 1.02 (0.68-1.52) | |
| Fracture of the shoulder/upper arm | 1; 6 | 931.5; 2553.6 | 1.1; 2.3 | 0.50 (0.05-4.91) |  | 36; 141 | 8113.8; 23653.4 | 4.4; 6.0 | 0.76 (0.51-1.13) | |
| Fracture of the forearm | 2; 7 | 931.5; 2553.6 | 2.1; 2.7 | 0.88 (0.18-4.42) |  | 57; 139 | 8113.8; 23653.4 | 7.0; 5.9 | 1.23 (0.85-1.76) | |
| Fracture of the pelvis | 3; 11 | 931.5; 2553.6 | 3.2; 4.3 | 0.75 (0.19-2.96) |  | 39; 79 | 8113.8; 23653.4 | 4.8; 3.3 | 1.53 (0.99-2.36) | |
| Fracture of the lower leg | 4; 6 | 931.5; 2553.6 | 4.3; 2.3 | 1.73 (0.49-6.13) |  | 26; 96 | 8113.8; 23653.4 | 3.2; 4.1 | 0.80 (0.48-1.33) | |
| **Age** | **≤ 78.5 years** | | | |  | **> 78.5 years** | | | |  |
| Fragilityfractures combined* | 136; 392 | 4416.0; 12560.4 | 30.8; 31.2 | 1.02 (0.79-1.31) |  | 177; 619 | 4629.3; 13646.6 | 38.2; 45.4 | 0.89 (0.73-1.09) | |
| Fracture of the femur | 39; 131 | 4416.0; 12560.4 | 8.8; 10.4 | 0.86 (0.59-1.25) |  | 51; 226 | 4629.3; 13646.6 | 11.0; 16.6 | 0.69 (0.50-0.97) | |
| Fracture of the spine | 20; 58 | 4416.0; 12560.4 | 4.5; 4.6 | 1.01 (0.56-1.84) |  | 35; 111 | 4629.3; 13646.6 | 7.6; 8.1 | 0.99 (0.63-1.56) | |
| Fracture of the shoulder/upper arm | 20; 63 | 4416.0; 12560.4 | 4.5; 5.0 | 0.92 (0.52-1.62) |  | 17; 84 | 4629.3; 13646.6 | 3.7; 6.2 | 0.63 (0.35-1.10) | |
| Fracture of the forearm | 26; 63 | 4416.0; 12560.4 | 5.9; 5.0 | 1.22 (0.71-2.09) |  | 33; 83 | 4629.3; 13646.6 | 7.1; 6.1 | 1.23 (0.79-1.92) | |
| Fracture of the pelvis | 12; 26 | 4416.0; 12560.4 | 2.7; 2.1 | 1.37 (0.67-2.8) |  | 30; 64 | 4629.3; 13646.6 | 6.5; 4.7 | 1.43 (0.89-2.30) | |
| Fracture of the lower leg | 19; 51 | 4416.0; 12560.4 | 4.3; 4.1 | 1.07 (0.59-1.95) |  | 11; 51 | 4629.3; 13646.6 | 2.4; 3.7 | 0.64 (0.31-1.31) | |
| **History of fracture^†^** | **No previous fracture** | | | |  | **Previous fracture** | | | |  |
| Fragility fractures combined* | 164; 543 | 6398.0; 18744.3 | 25.6; 29.0 | 0.94 (0.76-1.16) |  | 149; 468 | 2647.3; 7462.7 | 56.3; 62.7 | 0.92 (0.73-1.15) | |
| Fracture of the femur | 47; 184 | 6398.0; 18744.3 | 7.3; 9.8 | 0.78 (0.55-1.10) |  | 43; 173 | 2647.3; 7462.7 | 16.2; 23.2 | 0.72 (0.48-1.07) | |
| Fracture of the spine | 21; 74 | 6398.0; 18744.3 | 3.3; 3.9 | 0.89 (0.52-1.49) |  | 34; 95 | 2647.3; 7462.7 | 12.8; 12.7 | 1.04 (0.63-1.70) | |
| Fracture of the shoulder/upper arm | 21; 91 | 6398.0; 18744.3 | 3.3; 4.9 | 0.69 (0.42-1.15) |  | 16; 56 | 2647.3; 7462.7 | 6.0; 7.5 | 0.80 (0.45-1.43) | |
| Fracture of the forearm | 40; 89 | 6398.0; 18744.3 | 6.3; 4.7 | 1.30 (0.85-2.00) |  | 19; 57 | 2647.3; 7462.7 | 7.2; 7.6 | 1.03 (0.59-1.82) | |
| Fracture of the pelvis | 19; 46 | 6398.0; 18744.3 | 3.0; 2.5 | 1.31 (0.72-2.39) |  | 23; 44 | 2647.3; 7462.7 | 8.7; 5.9 | 1.49 (0.85-2.62) | |
| Fracture of the lower leg | 16; 59 | 6398.0; 18744.3 | 2.5; 3.1 | 0.81 (0.42-1.53) |  | 14; 43 | 2647.3; 7462.7 | 5.3; 5.8 | 0.94 (0.49-1.77) | |

* ICD-10 S12, S22, S32, S42, S52, S72, S82 combined

^†^ in the time period of 5 years before study entry; *Numbers of fractures per 1000 person-years;
IG: intervention group; CG control group
